# Computer Modeling of Clot Retrieval—Circle of Willis

**DOI:** 10.3389/fneur.2020.00773

**Published:** 2020-08-07

**Authors:** Thanh G. Phan, Henry Ma, Mayank Goyal, James Hilton, Matthew Sinnott, Velandai Srikanth, Richard Beare

**Affiliations:** ^1^Stroke and Aging Research Group, Clinical Trials, Imaging and Informatics Division, School of Clinical Sciences at Monash Health, Monash University, Melbourne, VIC, Australia; ^2^Department of Neurology, Monash Health, Melbourne, VIC, Australia; ^3^Departments of Clinical Neuroscience and Radiology, Cummings School of Medicine, Hotchkiss Brain Institute, University of Calgary, Calgary, AB, Canada; ^4^Data 61, CSIRO, Innovation Hub, Docklands, VIC, Australia; ^5^Department of Medicine, Peninsula Clinical School, Central Clinical School, Frankston Hospital, Monash University, Melbourne, VIC, Australia

**Keywords:** circle of Willis, leptomeningeal anastomoses, thrombectomy, simulation, stroke, carotid endarterectomy, angioplasty, endovascular clot retrieval

## Abstract

Endovascular clot retrieval, often referred to as mechanical thrombectomy, has transformed the treatment of patients with ischemic stroke based on an underlying large cerebral vessel occlusion, ranging from the extracranial internal carotid artery (ICA) to the M1 (proximal) segment of the middle cerebral artery (MCA). The aim of this study was to evaluate the effect of a progressive occlusion of the extracranial portion of the ICA on the cerebral blood flow either with a conventional guiding catheter or a balloon-guiding catheter, which enables the operator to completely occlude the parent artery by inflating the balloon around the tip of this type of guiding catheter. We evaluated the impact of flow reduction in the ICA in the setting of ipsilateral MCA occlusion given the different configurations of the circle of Willis (CoW). The computer model of cerebral arteries was based on anatomical works by Rhoton (1) and van der Eecken (2). The interactive experimental results are available on the web at https://gntem3.shinyapps.io/ecrsim. In the setting of left MCA occlusion, compensation from the anterior and posterior communicating artery preserved the flow in the left anterior cerebral artery (ACA) but not the left MCA branches. Under selected CoW configurations, such as classic, missing Acom, or missing A1 segment of the ACA and concurrent right ICA occlusion, there was a progressive decrease of flow in the left ACA to a minimum of 78% when the simulated catheter fully occluded the left ICA. Flow collapsed (<10%) in the left ACA and MCA branches under CoW configurations, such as bilateral fetal PCA. In summary, compensatory flow collapsed under certain clot retrieval scenarios and unusual configurations of CoW.

## Introduction

Endovascular clot retrieval (ECR) or mechanical thrombectomy transformed treatment of patients with large vessel occlusion (LVO) (3–8). Patients with LVO involving the internal carotid artery (ICA) and middle cerebral artery (MCA) or combined lesions have been successfully treated in these trials. There are still several unanswered questions with clot retrieval: safety of advancing catheter along ICA in setting of ipsilateral MCA occlusion, given the concomitant occlusion of the contralateral ICA. In this setting, there is a hypothetical possibility that the cross-flow across anterior communicating artery (Acom) and the partial protection provided by leptomeningeal anastomoses (LAs) would be insufficient. This question is hard to answer *in vivo* and can be investigated using a computer simulation model. This study uses a computer model, based on the anatomical works of Rhoton (1) and van der Eecken (2), which was created in our earlier work (9).

The circle of Willis (CoW) is made up of the ICA, the Acom and posterior communicating arteries (Pcom) and the proximal part of the anterior (ACA) and the proximal part of the posterior cerebral arteries (PCA). The MCA exists distal or downstream from the CoW. The CoW network (primary collateral system) protects the brain from the unilateral extracranial ICA occlusion as long as cross-flow within the CoW is not impeded (10–12). However, the CoW may not offer sufficient protection when the site of occlusion is in the MCA or its cortical arterial branches. Flow in the Pcom is usually in the anterior to posterior direction except when there is ICA occlusion (9, 13, 14). On magnetic resonance (MR) angiography, a complete CoW occurs in 36% of normal subjects and 55% of subjects with ICA occlusion (13). Similarly, a complete posterior CoW configuration was observed in 63% with ICA occlusion vs. 47% in normal subjects.

In many studies, the term *collateral anastomoses/circulation* is used without further specification. In this study, we used the term *leptomeningeal anastomosis* to describe a secondary collateral system and that exists between selected branches of the MCA, PCA, and ACA (2). The collaterals described on computed tomography angiography after LVO are likely to be due to the work of the LA in conjunction with CoW (15, 16). The contribution of this secondary collateral system has been observed in maps of infarct territory as relative sparing of the posterior and superior parts of the MCA territory (17, 18) and in our earlier work on the computer model of the cerebral circulation (9).

Our earlier work on the computer model of the cerebral circulation was based on a classic configuration with complete CoW (9). In this study, we have extended the model and incorporate other CoW configurations, as well as simulated catheter movement along the ICA. The aim of this study was to evaluate the effect of a progressive occlusion of the extracranial portion of the ICA on the cerebral blood flow either with a conventional guiding catheter or a balloon-guiding catheter, which enables the operator to completely occlude the parent artery by inflating the balloon around the tip of this type of guiding catheter. We evaluated the impact of flow reduction in the ICA in the setting of ipsilateral MCA occlusion given the different configurations of the CoW.

## Materials and Methods

### Computational Modeling

A network of cylindrical pipes was used to model blood flow within the brain. In the original publication, we created a two-dimensional model of the major cerebral arteries using MATLAB version 5 (The Mathworks Inc., Natick, MA, USA) (9). In this study, we have transformed the MATLAB codes into R codes (R Statistical Foundation) and added the different configurations of the CoW into the model. The model of the cerebral circulation was based on the anatomical works by Rhoton (1) and van der Eecken (2). The named branches of the MCA, ACA, and PCA were empirically drawn down to the fifth branching order. Connections between the fifth-order branches were used to represent the LA. Consistent with previous anatomical descriptions, interhemispheric connections by LA from ACA branches were permitted (1). This model was then converted to a list of nodes and pipes in graph theory package *igraph* in R (19).

We assumed that flow was laminar within the pipe network representing cerebral arteries and formulated a linked set of equations for the system by imposing mass balance and pressure (energy) balance over the network. Inflow and outflow boundary conditions were imposed on the model. We modeled flow in each of the ICA and the basilar artery (BA) [via the vertebral arteries] as coming off the aortic arch and heart (connection below the CoW). Based on previous works (20, 21), the inflow conditions were set so that 75% of the total flow passed through the two ICAs and 25% through the BA, and a pressure condition of 5 kPa was imposed over the outer boundary. The outflow was modeled as a consequence of the arterial network branching to smaller and smaller capillaries. We included a drainage component at end points of the arterial branches to accommodate this outflow. The set of equations governing volume, flow rate, and pressure over the whole network was solved iteratively. The process was repeated for several different experiments in which successive arteries or combinations of arteries were occluded in the anterior circulation.

### Primary Outcome

The primary outcome was maintenance of flow above a theoretical threshold (defined as 30% of baseline flow) in the setting of LVO (22). Baseline flow was defined according to flow on the contralateral side. This definition of ischemic core (30% of relative blood flow to the normal contralateral side) has also been used in clinical trials (23).

### Experiments

There are two key experiments:

(1) occlusion of the right ICA and left MCA trunk or branches and
(2) occlusion of left MCA trunk or branches.

### Catheter

For each experiment, we simulated the movement of a catheter along the artery or balloon occlusion by imposing stenosis in the ICA. The sensitivity analysis was performed with stenosis ranging from 0, 25, 50, 75, 90, 99, and 100%.

### Leptomeningeal Anastomoses

Each experiment was performed with at different interterritory LA. We explored the effect of augmentation of LA by setting the diameter of LA between 0.5 and 1.5 mm. The default LA size was 1 mm in these experiments (9).

**Figure 1:**
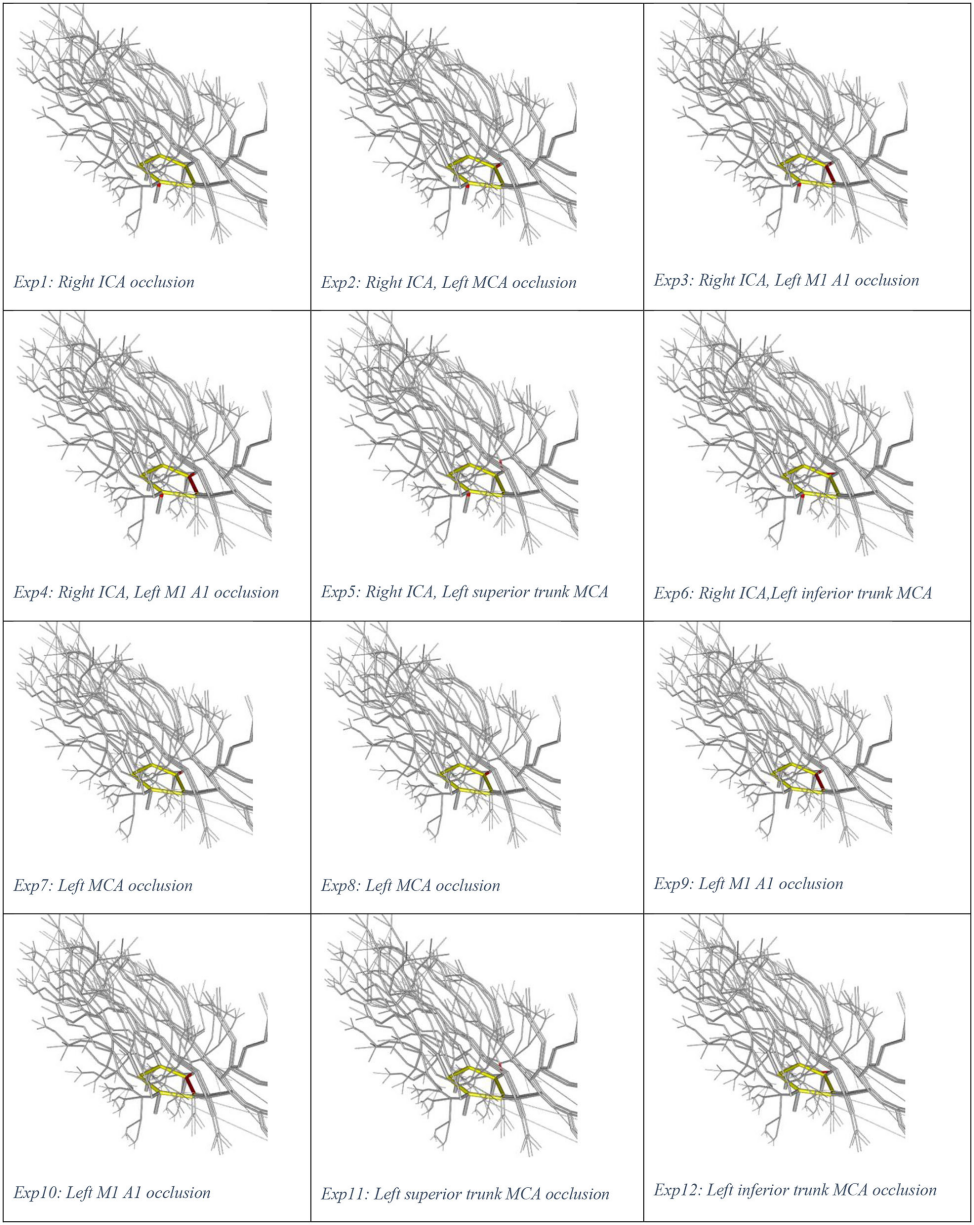
Visual representations of Experiments 1–12.

Illustrations of the experiments are presented in Figure 1. Experiment 0 is not displayed as there is no occlusion. We used the plotting library *plotly* in R to create a video play loop in which each frame of the video displays a single experiment. These experiments were combined so that the viewer can move through them with a “feel” of a movie. We have also collated the change in ICA stenosis into a movie, with each frame representing the degree of stenosis. These results are available at https://gntem3.shinyapps.io/ecrsim. The web-based app contains four tabs. The first tab, “Moving site of occlusion,” is for exploring CoW configurations, experiments, and LA diameter. The second tab, “Catheter causing progressive stenosis,” allows manipulation of the CoW configuration, degree of stenosis, and LA diameter. The third tab, “Artery model,” displays the three dimensional model of cerebral artery. The fourth tab, “Data table,” allows the viewers to peruse the results in a table format.

## Results

### Complete CoW

**Figure 2:**
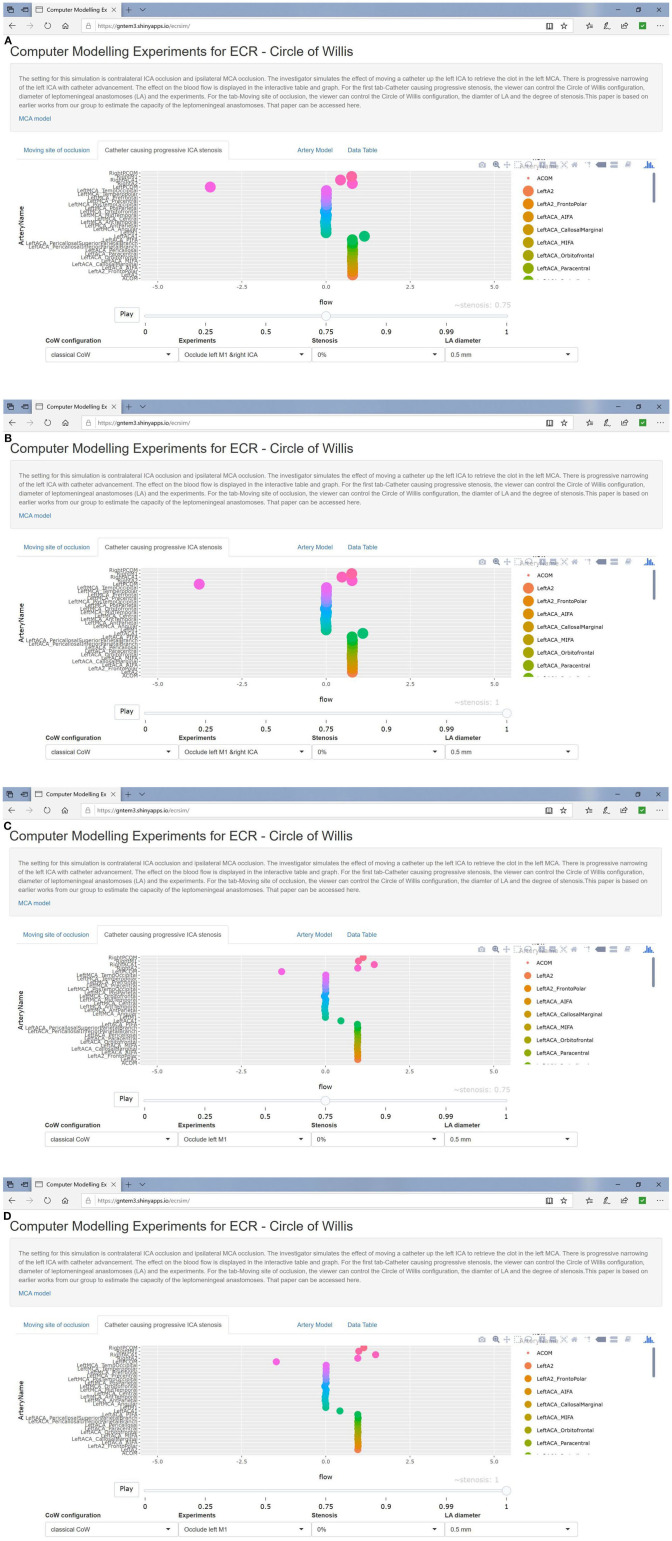
**(A,B)** Simulated catheter movement reduced flow to 86% of baseline in left ACA branches in setting of contralateral ICA occlusion. In **(C,D)**, catheter movement has no effect even at 100% stenosis (absence of contralateral ICA occlusion).

In the setting of left MCA occlusion, there was recruitment of the CoW vessels (Acom and left Pcom). This scenario resulted in compensatory cross flow as well as flow from the posterior circulation into the proximal left ACA and its branches, but not the MCA branches (see Table 1 and full results at https://gntem3.shinyapps.io/ecrsim). Augmentation of LA diameter improved flow in selected arteries, such as left posterior parietal, from 0% at LA = 0.5 mm to 13% at LA = 1 mm to 23% at LA = 1.5 mm. Even with increased LA diameters, there were no changes in flow in some arteries, such as the precentral (1%) and central (3%) arteries. These findings were also observed in the rare setting of concurrent left MCA occlusion and right ICA occlusion. Progressive stenosis of the ipsilateral ICA between 0 and 50% did not affect flow in the left ACA. At 75% ICA stenosis and above, flow in the left ACA branches dropped to 88%. At 100% stenosis, flow in the left ACA branches dropped to 86% (Figure 2).

**Table 1:** Flow in different circle of Willis configurations.

**No left ICA occlusion**	**Classic**	**Acom missing**	**Left A1 missing**	**Left A1 P1 missing**	**Bilateral P1 missing**	**Bilateral P1 and left A1 missing**
Left ACA–paracentral artery	100	100	94	90	93	90
Left MCA–posterior parietal artery	100	100	104	96	93	96
Acom	100	NA	30,325	28,992	83	27,478
Left Pcom	100	100	216	457	441	457
**LEFT ICA OCCLUSION**						
Left ACA–paracentral artery	90	80	99	102	77	102
Left MCA–posterior parietal artery	86	79	84	9	66	8
Acom	32,970	NA	99	102	92,518	102
Left Pcom	−386	−648	−199	1	64	1

### Absent Acom

**Figure 3:**
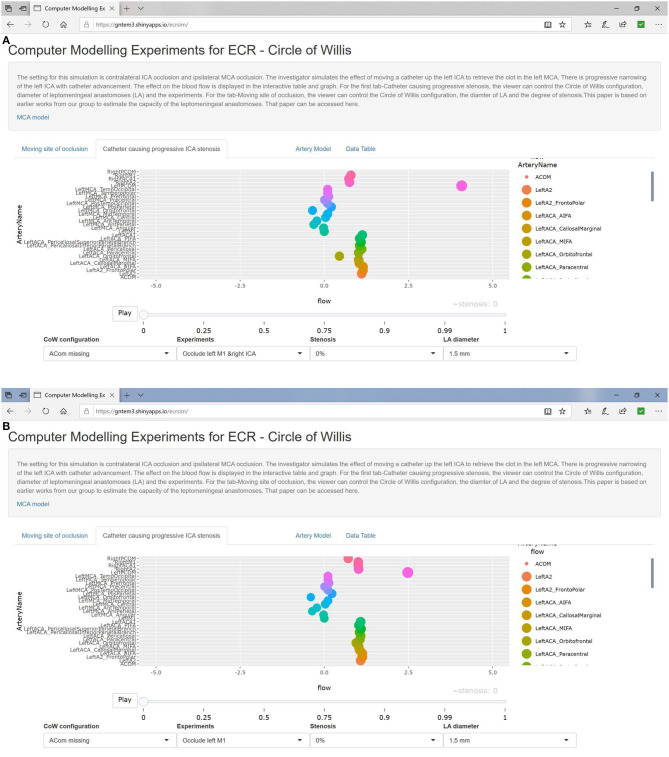
**(A)** Left MCA occlusion and contralateral ICA occlusion. **(B)** Left MCA occlusion only. Catheter movement has no effect even at 100% stenosis (absence of contralateral ICA occlusion).

Flow was reduced in the left ACA (77%) and MCA (75%) in the presence of 100% left ICA occlusion. The additional left MCA (to left ICA) occlusion resulted in flow dropping below 12% in the left MCA branches and to 87% of baseline flow in the left ACA branches. We created T junction occlusion of the ICA by occluding the left MCA, ICA, and A1. In this experiment, flow in the left MCA branches dropped below ischemic threshold (12%), but flow in the left ACA branches was preserved. The same result occurred in the setting of complete CoW, as the left ACA has support from the right ICA via the Acom (Figure 3). In the setting of left MCA occlusion and contralateral right ICA occlusion, flow was reduced below ischemic threshold in the left MCA branches (<12%) and minimal changes in the left ACA branches (93%). In this scenario, stenosis of the ipsilateral (left) ICA between 0 and 100% did not further reduce flow in the left MCA branches, and there was only a small reduction in flow in the left ACA branches.

### Absent Left A1 Segment

In the setting of left MCA occlusion, there were reduced flow in the left MCA branches below ischemic threshold (<12%) and minimal changes in the left ACA branches (98%). In the setting of left MCA and right ICA occlusion, there was reduced flow in the left MCA branches below ischemic threshold (<12%) and a smaller reduction in flow in the left ACA branches (78%). Augmenting interterritory LA from 0.5 to 1.5 mm did not result in any change in flow.

### Absent Left A1 and Left Fetal PCA

The findings were similar to the experiment with absent left A1 configuration of CoW. Augmentation of the LA from 0.5- to 1.5-mm diameter made no change to flow in the left ACA branches.

### Bilateral Fetal PCA

**Figure 4:**
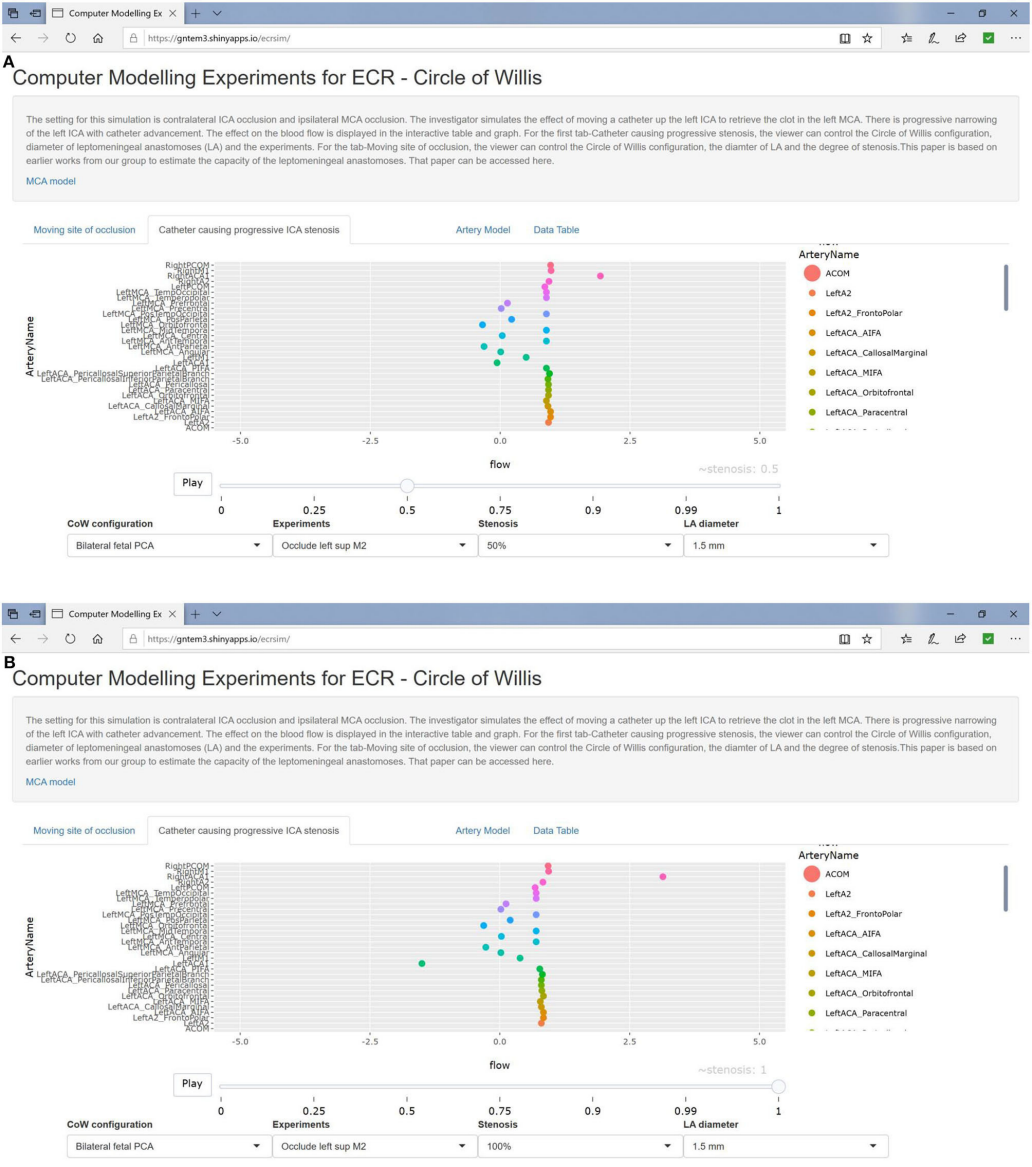
Simulated catheter movement on the left ICA show mild reduction in flow in the MCA branches at 50% stenosis **(A)** and at 100% stenosis **(B)**.

In the setting of left MCA occlusion and contralateral (right) ICA occlusion, there was preserved flow in the ACA branches and recruitment of the CoW vessels (doubling of flow in the left Pcom) at low stenosis of the left ICA. Flow in Pcom completely collapsed as the stenosis in the ICA increased (≥50%) (Figure 4). Augmentation of the LA to 1.5-mm diameter made no change to flow in the left ACA branches.

### Bilateral Fetal PCA and Absent Left A1

In the setting of left MCA and contralateral right ICA occlusion, there was no flow in the ACA branches even at no stenosis of the left ICA. Augmentation of the LA to 1.5-mm diameter made no change to flow in the left ACA branches.

### Balloon Occlusion of Left ICA

Progressive stenosis (reduction of diameter) of left ICA did not result in any change in CoW flow until 50% occlusion (left MCA branches <91% and left ACA branches <95%). At 100% left ICA stenosis, flow was reduced in the left MCA branches <84% and left ACA branches <90%. In the setting of absent left A1, there was reduced flow in the left ACA branches (94%), mildly increased flow in the left MCA branches (104%), and markedly increased flow in the left Pcom (215%). In the setting of absent left A1 and P1, there was reduced flow in left MCA branches (9%). The changes were marked in the presence of CoW configuration with bilateral fetal PCA and absent left A1, where there was no flow in the left ACA branches.

In the presence of right ICA occlusion, progressive stenosis of the left ICA led to reduced flow in the left ACA (90%) and MCA branches (86%). With absent Acom, flows in the left ACA and MCA were reduced to 80 and 79%, respectively. With absent left A1, flows in the left ACA branches were reduced to 99%, and MCA branches to 84%. When both the left A1 and P1 were absent, flow in the left ACA was maintained at 102% and reduced in the left MCA to 9%. With bilateral fetal PCA and absent left A1, flow was maintained in the left ACA (102%) and reduced in the left MCA branches (8%).

## Discussion

In this study, we have utilized the capability of a computer model of the cerebral arteries to explore the effect of advancing a catheter for clot retrieval under different hypothetical LVO scenarios. The simulations show that, under selected configurations of CoW (such as absent Acom or bilateral fetal PCA pattern), there is potential for a collapse of cerebral blood flow. We have animated the experiments to illustrate the dynamic process of catheter movement or balloon occlusion. Further, these experiments have been uploaded onto the web so that others are able to explore these hypothetical LVO scenarios interactively.

### CoW Configuration

In this study, we corrected a limitation of the earlier model of the cerebral circulation, which only used a complete CoW configuration (9). The large range of CoW configurations used in this study reflected the different scenarios seen in clinical practice. Surprisingly, information on the individual configurations of the CoW in the cohorts of the randomized controlled ECR trials has not yet been fully published (3–8). The focus in secondary analyses of these pivotal trials has been on the leptomeningeal collateral system (15, 16). A secondary analysis of the data from the first positive multicentric randomized clinical trial of endovascular treatment for acute ischemic stroke (MRCLEAN) from the Netherlands observed smaller thrombi in patients with patent Pcom and ACA (16). In summary, these studies have not yet discussed the potential implications of different, individual CoW configurations on success or failure of ECR. There have also been studies using silicone-based phantoms of arterial trees (24, 25). These phantoms were built to test (endovascular) medical devices and for learning rather than to evaluate the circulation under different CoW configurations and LVO scenarios. One group performed physical simulation of the CoW with different degrees of ICA stenosis but, in contrast to our study, did not include LA collaterals in the model (26); they reported that configurations of the CoW with absent Pcom might be critical in terms of stroke outcome, based on their *in vitro* experiment. Furthermore, the collateral capacity of the Pcom became more important as ICA stenosis rose above 40%.

### Simulation of Catheter Movement

There are sparse data in the literature on the effect of advancing a catheter along the ICA for clot retrieval under different CoW configurations. Investigators have discussed the impact of different types of ICA occlusions on collateral flow. The various ICA occlusions are as follows: I type (ICA occlusion), L type (ICA and MCA occlusion), and T type (ICA, MCA, and ACA occlusion) (27). However, there was no detailed discussion of the CoW in this article or in a more recent review on the topic of collateral circulation in the setting of thrombectomy (27, 28). One article described the importance of Acom and Pcom to the existence of LA collaterals (29). A similar scenario may occur during the performance of carotid endarterectomy in the setting of contralateral ICA occlusion. Carotid endarterectomy requires clamping of the ipsilateral ICA to achieve hemostasis and prevention of distal embolization. This procedure, but not angioplasty and stenting, has been associated with increased risk of stroke in the setting of contralateral carotid occlusion (30, 31). Unfortunately, there was no information on the status of the CoW in these studies. This is a subject that we hope would change in the future as investigators reevaluate the impact of CoW configurations on procedure feasibility and outcome.

To address the paucity of data on this topic, we imposed different degrees of ICA stenosis to simulate the effect of catheter advancement in the ICA or balloon inflation. These experiments were performed to evaluate downstream flow in the ACA and MCA branches. Our results suggested that under certain unfavorable CoW configurations, such as ipsilateral absent A1 and fetal PCA, there was collapse of the collateral systems. The results of these experiments are difficult to replicate in humans and would pose ethical issues given the findings here. Some support for our findings is provided by earlier studies in which there were descriptions of the use of manual techniques or balloon-guided compression of the ICA to evaluate safety of ICA occlusion for treatment of aneurysm (32–36). These studies surprisingly did not describe the CoW configuration before and after occlusion. A more recent article suggested the importance of cross-flow across the Acom and that its absence would result in a positive balloon occlusion test (37). Nikoubashman et al. have shown that a large-bore catheter can reduce, but not arrest, flow in the parent vessel in a porcine model (38). Complete flow arrest and flow reversal during clot retrieval, however, can be achieved by using a balloon-guided catheter and distal aspiration catheter (so-called dual aspiration).

### Leptomeningeal Anastomoses

The size of the interterritorial LA in these experiments did not modify flow patterns in critical arteries when paired with unfavorable CoW configurations. As such, augmentation of LA may not improve flow when the proximal source of flow had been interrupted. Our original experiments were done with complete CoW configuration, where provision of flow to the left ACA branches and onto the selected MCA branches was possible (9).

### Limitations

An issue with the earlier work on the computer model of cerebral circulation was that there was no method developed for depicting the results. We have attempted to rectify this issue by creating an app for exploring CoW configurations, ICA stenosis, arterial occlusion, and augmentation of LA. A potential drawback of this approach is that the reader needs to simultaneously read the article and explore the app. The “Artery model” tab can take several minutes to load on the web with Microsoft Edge. Loading this page is faster when using Google Chrome and fastest with Firefox on Linux operating system.

The experiments were created using a graph theory library (*igraph*) rather than the more computationally expensive physical model of the cerebral arteries with exact three-dimensional geometry. We are not aware of such a model at this stage and that would allow for this type of modeling or viewing. There are several groups who have described probabilistic map of the cerebral anatomy based on MR angiography performed on 3- and 7-T scanners (39–41). These atlases have labeling of the CoW, MCA, ACA, and PCA but not the major branches of these arteries and the LA. By contrast, this computer model of the cerebral arteries has labeling of the arterial branches down to the fifth order. A more rigorous method would have been to segment and label different segments of the entire cerebral circulation from whole-brain radiological images at a minimum on 7-T MR scanner; this is an enormous and currently infeasible task. Given the current technology, acquisition of micromillimeter high-resolution imaging of the LA and segmentation and labeling of these arteries are not yet possible. The challenge is less important in the context of our study because we did not intend to study fluid hemodynamics, but rather simply to examine the reserve capacity of the CoW and LA.

This simulation study showed that configurations of CoW play a major role in determining outcome. Augmentation of the leptomeningeal system is not sufficient in this setting to change blood flow to >30% of baseline. We hope that this study will lead to evaluation of the configurations of CoW in the ECR trials to better inform clinicians when faced with these clinical scenarios.

## Data Availability Statement

The raw data supporting the conclusions of this article will be made available by the authors, without undue reservation.

## Author Contributions

TP: design, creation of app to display result, analysis, and writing of manuscript. RB: transfer original Matlab script into R, depiction of computer model analysis, creation of app to display result, and writing of manuscript. JH and MS: Matlab script for computer model. MG, VS, and HM: writing of manuscript. All authors contributed to the article and approved the submitted version.

## Conflict of Interest

TP has received honoraria as speaker for Bayer, Boehringer Ingelheim, Sanofi-Genzyme. He serves on the advisory board for Sanofi received-Genzyme for Fabry Disease. The remaining authors declare that the research was conducted in the absence of any commercial or financial relationships that could be construed as a potential conflict of interest.

## Abbreviations

ACA: anterior cerebral artery
Acom: anterior communicating artery
BA: basilar artery
CoW: circle of Willis
ECR: endovascular clot retrieval
ICA: internal carotid artery
LA: leptomeningeal anastomoses
LVO: large vessel occlusion
MCA: middle cerebral artery
MR: magnetic resonance
MRCLEAN: multicenter randomized clinical trial of endovascular treatment for acute ischemic stroke in the Netherlands
PCA: posterior cerebral artery
Pcom: posterior communicating artery.

## References

[1] Rhoton AL Jr The supratentorial arteries. Neurosurgery. (2002) 51:S53–120. 10.1097/00006123-200210001-00003 12234447

[2] Van der Eecken HM The Anastomoses Between the Leptomeningeal Arteries of the Brain: Their Morphological, Pathological and Clinical Significance. Springfiled, IL: Charles C. Thomas (1959).

[3] Berkhemer OA Fransen PS Beumer D van den Berg LA Lingsma HF Yoo AJ. A randomized trial of intraarterial treatment for acute ischemic stroke. N Engl J Med. (2015) 372:11–20. 10.1056/NEJMoa1411587 25517348

[4] Goyal M Menon BK van Zwam WH Dippel DW Mitchell PJ Demchuk AM. Endovascular thrombectomy after large-vessel ischaemic stroke: a meta-analysis of individual patient data from five randomised trials. Lancet. (2016) 387:1723–31. 10.1016/S0140-6736(16)00163-X 26898852

[5] Goyal M Demchuk AM Menon BK Eesa M Rempel JL Thornton J. Randomized assessment of rapid endovascular treatment of ischemic stroke. N Engl J Med. (2015) 372:1019–30. 10.1056/NEJMoa1414905 25671798

[6] Jovin TG Chamorro A Cobo E de Miquel MA Molina CA Rovira A. Thrombectomy within 8 hours after symptom onset in ischemic stroke. N Engl J Med. (2015) 372:2296–306. 10.1056/NEJMoa1503780 25882510

[7] Campbell BC Mitchell PJ Kleinig TJ Dewey HM Churilov L Yassi N. Endovascular therapy for ischemic stroke with perfusion-imaging selection. N Engl J Med. (2015) 372:1009–18. 10.1056/NEJMoa1414792 25671797

[8] Saver JL Goyal M Bonafe A Diener HC Levy EI Pereira VM. Stent-retriever thrombectomy after intravenous t-pa vs. T-pa alone in stroke. N Engl J Med. (2015) 372:2285–95. 10.1056/NEJMoa1415061 25882376

[9] Phan TG Hilton J Beare R Srikanth V Sinnott M. Computer modeling of anterior circulation stroke: proof of concept in cerebrovascular occlusion. Front Neurol. (2014) 5:176. 10.3389/fneur.2014.00176 25285093 PMC4168699

[10] Brozici M van der Zwan A Hillen B. Anatomy and functionality of leptomeningeal anastomoses: a review. Stroke. (2003) 34:2750–62. 10.1161/01.STR.0000095791.85737.65 14576375

[11] Cassot F Vergeur V Bossuet P Hillen B Zagzoule M Marc-Vergnes JP Effects of anterior communicating artery diameter on cerebral hemodynamics in internal carotid artery disease. A model study. Circulation. (1995) 92:3122–31. 10.1161/01.CIR.92.10.3122 7586284

[12] Schomer DF Marks MP Steinberg GK Johnstone IM Boothroyd DB Ross MR. The anatomy of the posterior communicating artery as a risk factor for ischemic cerebral infarction. N Engl J Med. (1994) 330:1565–70. 10.1056/NEJM199406023302204 8177246

[13] Hartkamp MJ van Der Grond J van Everdingen KJ Hillen B Mali WP. Circle of willis collateral flow investigated by magnetic resonance angiography. Stroke. (1999) 30:2671–78. 10.1161/01.STR.30.12.2671 10582995

[14] Hendrikse J Hartkamp MJ Hillen B Mali WP van der Grond J. Collateral ability of the circle of willis in patients with unilateral internal carotid artery occlusion: Border zone infarcts and clinical symptoms. Stroke. (2001) 32:2768–73. 10.1161/hs1201.099892 11739971

[15] Berkhemer OA Jansen IG Beumer D Fransen PS van den Berg LA Yoo AJ. Collateral status on baseline computed tomographic angiography and intra-arterial treatment effect in patients with proximal anterior circulation stroke. Stroke. (2016) 47:768–76. 10.1161/STROKEAHA.115.011788 26903582

[16] Alves HC Treurniet KM Dutra BG Jansen IGH Boers AMM Santos EMM. Associations between collateral status and thrombus characteristics and their impact in anterior circulation stroke. Stroke. (2018) 49:391–6. 10.1161/STROKEAHA.117.019509 29321337

[17] Phan TG Donnan GA Srikanth V Chen J Reutens DC. Heterogeneity in infarct patterns and clinical outcomes following internal carotid artery occlusion. Arch Neurol. (2009) 66:1523–28. 10.1001/archneurol.2009.259 20008658

[18] Kim DE Park JH Schellingerhout D Ryu WS Lee SK Jang MU. Mapping the supratentorial cerebral arterial territories using 1160 large artery infarcts. JAMA Neurol. (2018) 76:72–80. 10.1001/jamaneurol.2018.2808 30264158 PMC6439879

[19] Csardi G Nepusz T The *igraph* software package for complex network research. Inter J. (2006) 1695:1–9.

[20] Scheel P Ruge C Petruch UR Schoning M. Color duplex measurement of cerebral blood flow volume in healthy adults. Stroke. (2000) 31:147–50. 10.1161/01.STR.31.1.147 10625730

[21] Heywood J Rannacher R Turek S Artificial boundaries and flux and pressure conditions for the incompressible navier-stokes equations. Int J Num Methods Fluids. (1996) 22:325–52. 10.1002/(SICI)1097-0363(19960315)22:5<325::AID-FLD307>3.0.CO;2-Y

[22] Hossmann KA. Viability thresholds and the penumbra of focal ischemia. Ann Neurol. (1994) 36:557–65. 10.1002/ana.410360404 7944288

[23] Campbell BCV Majoie C Albers GW Menon BK Yassi N Sharma G. Penumbral imaging and functional outcome in patients with anterior circulation ischaemic stroke treated with endovascular thrombectomy versus medical therapy: a meta-analysis of individual patient-level data. Lancet Neurol. (2019) 18:46–55. 10.1016/S1474-4422(18)30314-4 30413385

[24] Chueh JY Wakhloo AK Gounis MJ. Neurovascular modeling: small-batch manufacturing of silicone vascular replicas. AJNR Am J Neuroradiol. (2009) 30:1159–64. 10.3174/ajnr.A1543 19321626 PMC2695841

[25] Kaneko N Mashiko T Ohnishi T Ohta M Namba K Watanabe E. Manufacture of patient-specific vascular replicas for endovascular simulation using fast, low-cost method. Sci Rep. (2016) 6:39168. 10.1038/srep39168 27976687 PMC5156941

[26] Zhu G Yuan Q Yang J Yeo J. Experimental study of hemodynamics in the circle of Willis. Biomed Eng Online. (2015) 14:S10. 10.1186/1475-925X-14-S1-S10 25603138 PMC4306098

[27] Liebeskind DS Flint AC Budzik RF Xiang B Smith WS Duckwiler GR. Carotid i's, l's and t's: collaterals shape the outcome of intracranial carotid occlusion in acute ischemic stroke. J Neurointerv Surg. (2015) 7:402–7. 10.1136/neurintsurg-2014-011231 24789707 PMC4216639

[28] Piedade GS Schirmer CM Goren O Zhang H Aghajanian A Faber JE. Cerebral collateral circulation: A review in the context of ischemic stroke and mechanical thrombectomy. World Neurosurg. (2018) 122:33–42. 10.1016/j.wneu.2018.10.066 30342266

[29] Millesi K Mutzenbach JS Killer-Oberpfalzer M Hecker C Machegger L Bubel N Influence of the circle of willis on leptomeningeal collateral flow in anterior circulation occlusive stroke: friend or foe? J Neurol Sci. (2019) 396:69–75. 10.1016/j.jns.2018.11.002 30419369

[30] Gasecki AP Eliasziw M Ferguson GG Hachinski V Barnett HJ Long-term prognosis and effect of endarterectomy in patients with symptomatic severe carotid stenosis and contralateral carotid stenosis or occlusion: results from NASCET. North American Symptomatic Carotid Endarterectomy Trial (NASCET) group. J Neurosurg. (1995) 83:778–82. 10.3171/jns.1995.83.5.0778 7472542

[31] Antoniou GA Kuhan G Sfyroeras GS Georgiadis GS Antoniou SA Murray D. Contralateral occlusion of the internal carotid artery increases the risk of patients undergoing carotid endarterectomy. J Vasc Surg. (2013) 57:1134–45. 10.1016/j.jvs.2012.12.028 23462196

[32] Linskey ME Jungreis CA Yonas H Hirsch WL Jr Sekhar LN Horton JA. Stroke risk after abrupt internal carotid artery sacrifice: accuracy of preoperative assessment with balloon test occlusion and stable xenon-enhanced ct. AJNR Am J Neuroradiol. (1994) 15:829–43. 8059649 PMC8332190

[33] van Rooij WJ Sluzewski M Slob MJ Rinkel GJ. Predictive value of angiographic testing for tolerance to therapeutic occlusion of the carotid artery. AJNR Am J Neuroradiol. (2005) 26:175–8. 15661722 PMC7975014

[34] Sato K Shimizu H Inoue T Fujimura M Matsumoto Y Kondo R. Angiographic circulation time and cerebral blood flow during balloon test occlusion of the internal carotid artery. J Cereb Blood Flow Metab. (2014) 34:136–43. 10.1038/jcbfm.2013.176 24103905 PMC3887353

[35] Mathis JM Barr JD Jungreis CA Yonas H Sekhar LN Vincent D. Temporary balloon test occlusion of the internal carotid artery: experience in 500 cases. AJNR Am J Neuroradiol. (1995) 16:749–54. 7611033 PMC8332245

[36] Imai K Mori T Izumoto H Takabatake N Kunieda T Shimizu H. Clot removal therapy by aspiration and extraction for acute embolic carotid occlusion. AJNR Am J Neuroradiol. (2006) 27:1521–27. 16908572 PMC7977536

[37] Kikuchi K Yoshiura T Hiwatashi A Togao O Yamashita K Honda H. Balloon test occlusion of internal carotid artery: angiographic findings predictive of results. World J Radiol. (2014) 6:619–24. 10.4329/wjr.v6.i8.619 25170400 PMC4147443

[38] Nikoubashman O Wischer D Hennemann HM Sandmann J Sichtermann T Muschenich FS. Balloon-guide catheters are needed for effective flow reversal during mechanical thrombectomy. AJNR Am J Neuroradiol. (2018) 39:2077–81. 10.3174/ajnr.A5829 30309845 PMC7655356

[39] Wright SN Kochunov P Mut F Bergamino M Brown KM Mazziotta JC. Digital reconstruction and morphometric analysis of human brain arterial vasculature from magnetic resonance angiography. Neuroimage. (2013) 82:170–81. 10.1016/j.neuroimage.2013.05.089 23727319 PMC3971907

[40] Dunas T Wahlin A Ambarki K Zarrinkoob L Malm J Eklund A. A stereotactic probabilistic atlas for the major cerebral arteries. Neuroinformatics. (2017) 15:101–10. 10.1007/s12021-016-9320-y 27873151 PMC5306162

[41] Forkert ND Fiehler J Suniaga S Wersching H Knecht S Kemmling A. A statistical cerebroarterial atlas derived from 700 mra datasets. Methods Inform Med. (2013) 52:467–74. 10.3414/ME13-02-0001 24190179

